# Use of Interpersonal Counseling for Modern Type Depression

**DOI:** 10.1155/2017/9491348

**Published:** 2017-10-25

**Authors:** Hisae Ono, Ami Yamamoto, Reiko Taketani, Emi Tsujimoto

**Affiliations:** Department of Psychological Science, Graduate School of Humanities, Kwansei Gakuin University, 1-155 Uegahara Ichibancho, Nishinomiya, Hyogo 662-8501, Japan

## Abstract

A novel form of depression, called “modern type depression” (MTD), has been increasing in prevalence in Japan. Patients with MTD present with an overt appeal of depressive mood and a desire to be excused from their work duties; as such, this can cause considerable trouble in the workplace. Psychosocial interventions should be primarily considered for the treatment of MTD. Interpersonal counseling (IPC), which has proven effective for treating subthreshold depression, may be effective for MTD. However, IPC is rarely done in Japan. Herein, we report on a successful case of IPC for a woman in her thirties who was about to quit her job due to MTD (diagnosed by the criteria for research use). After IPC, the patient enjoyed good communication with her boss and continued her job without succumbing to her depression. This case suggests that IPC may be effective for MTD in workers and further highlights the benefits of teaching interpersonal communication methods in the workplace.

## 1. Introduction

Increasingly more primary care patients are presenting with a diagnosis of “new type depression” in Japan [1]. “New type depression” generally refers to a depressive state that is completely different from melancholic depression. However, “new type depression” is not a medical term, and the concept has been used ambiguously; thus, its use has caused confusion in primary care settings. Recently, operationally defined diagnostic criteria and interventions for “new type depression” have been published [2]. In these criteria, the disease was defined as “modern type depression” (MTD). These criteria were recommended for use in research. A recent study has also suggested that MTD that meets these criteria might exist as a condition not only in Japan but also in many other countries with a variety of sociocultural and historical backgrounds [3].

According to these research criteria, individuals with MTD exhibit the following clinical futures: an overt appeal of depressive mood based on their own beliefs, expressing a desire to be excused or spared from their duties or responsibilities because of their depression, and poorer overall functioning during work or school, whereas it is relatively higher than at other times. They also have some specific personality characteristics; namely, they are not diligent, exhibit an avoidance/hatred of hierarchies in society and a preference to exist without social roles, are extrapunitive, and tend to feel a vague sense of omnipotence. A typical case of MTD does not meet the criteria for major depressive disorder (MDD) according to the Diagnostic and Statistical Manual of Mental Disorders, Fourth Edition, Text Revision (DSM-IV-TR) [4]. Rather, many cases of MTD are considered to be mildly to moderately depressed. Severely depressed cases are uncommon.

There is no established treatment of MTD. In research on the condition, psychosocial interventions have been primarily considered. Pharmacotherapy might be effective, but unwarranted medication can prolong or worsen the condition, suggesting that it requires caution [2]. However, caution is necessary even when considering psychosocial interventions: some psychotherapies requiring self-insight, such as psychoanalysis, are often unacceptable to patients with MTD because these patients believe that they are not the ones who should change [5]. These factors make MTD resistant to treatment and highly likely to become chronic. This in turn leads to further deterioration in adaptation at work or school and may eventually lead to social withdrawal [6].

Interpersonal counseling (IPC) is a brief, structured psychological intervention directly derived from interpersonal psychotherapy, which is a time-limited, evidence-based form of psychotherapy commonly used to treat major depression [7]. IPC was originally designed for use in primary care by professionals not specialized in mental health to treat distressed patients whose symptoms derive from current life stress [8]. Importantly, it is not a form of psychotherapy. Rather, it is a form of counseling for people who generally do not require psychiatric treatment. It is characterized by sympathy and education; the counselor maintains unconditional warmth toward the client and does not give instructions or interrupt clients' expression of their personality or past. The aim of IPC is not to change the client's personality but rather to help the client escape from a temporary stressful situation [9].

IPC comprises an initial 60-minute session followed by five 20-minute sessions in principle [8, 9]. In the first session, the counselor builds a relationship with the client and judges whether the client is suitable for IPC. Subsequently, they enter into an IPC contract. In the second session, the counselor focuses on patients' current interpersonal problems and social functioning in four problem areas: “complicated grief,” “interpersonal disputes,” “role transitions,” and “interpersonal deficits.” “Complicated grief” becomes the object of focus when a stress reaction is related to the loss of an important person. “Interpersonal disputes” are focused on when the client is experiencing discordance with spouses, colleagues, and so forth, and focus is put on “role transitions” when the client is experiencing major lifestyle changes such as a new workplace. “Interpersonal deficits” are attended to when the client is experiencing social isolation. Typically, one or two of these problem areas are selected, and the sessions are then built around considering how to handle these situations. In the 3rd to 5th sessions, the counselor typically helps clients respond positively to the problem area(s) of focus. This typically involves the counselor attempting to clarify the problem and improve how the client responds to it by noticing aspects of the situation that the client might have overlooked. The counselor might propose homework and utilize role-play to accelerate this change process. In the 6th session, the counselor makes the client reflect on their progress in dealing with the stress and talks about the conclusion of IPC, encouraging the client to believe that they are capable of dealing with future problems. The IPC manual does note that there is flexibility in the number of sessions, merely suggesting that effective change may take more than two sessions. It further notes that sessions can be terminated once a client's transient problem has been resolved, and he/she needs or desires no further sessions [8, 9].

IPC might be effective for treating MTD. This is because MTD might respond best to psychosocial interventions, and IPC has proven effective for treating subthreshold depression in primary care [8]. Moreover, the distortion of interpersonal relations is considered one of the main pathologies of MTD [2], and psychotherapies that require self-insight are often considered unacceptable to people with MTD [5]. Indeed, these individuals often do not accept counseling when they feel that they are being in any way criticized. IPC might be easier for them to accept because of its educational elements (i.e., its focus on improving specific communication skills in interpersonal relationships) and because the counselor occasionally takes a supportive stance. It is also rather easy to perform in primary care and can be administered by nonmental health professionals, such as general physicians and nurses. A single session (except the first) takes only around 20 min, meaning that it can be done during usual daily practice.

However, at present, there are no reports of the effectiveness of IPC for MTD, except for a case report indicating that IPC was effective for an individual with so-called “new type depression” [10]. Therefore, in this paper, we report on how IPC was effective for a single patient with MTD. The study was approved by the Kwansei Gakuin University Institutional Regulations for Research with Human Participants, and the work was conducted according to the principles of the Declaration of Helsinki. To ensure the patient's anonymity, the case details have been changed somewhat, although the essential content has not.

## 2. Case Presentation

*Case*. The case of a woman in her early thirties is reported.

*Chief Complaint*. “Please write me a medical certificate of depression that allows me to get leave from my job.”

*Medical History*. The woman had no previous psychiatric disorder.

*Family History*. There was nothing of note.

*Developmental and Social History*. The woman exhibited no psychological problems during her junior and senior high school or university years. She often did not work hard on her studies but nevertheless experienced no notable problems. After graduating from university, she got a job without much of a struggle with job hunting. At that time, she was single and lived by herself. She described herself as a cheerful person, just like anyone else. Her attitude was suitable for her age, but her speech while conversing with the doctor was childish.

*History of Present Illness*. A month before she visited our clinic, she moved to a new workplace. She felt her new work was more difficult and that her new boss was “terrible.” Around that time, she began to feel ill and sought a full medical checkup, but no physical problems were found. She then self-diagnosed herself with depression based on Internet information and visited our primary care clinic, which handles cases related to psychosomatic and internal medicine.

*Present Illness*. At the first medical examination in our clinic, she said, “I want to quit my job. I cry when I try to go to work. I am suffering from depression called MDD. I have checked my symptoms on the Internet. Please write me a medical certificate that allows me to get leave from my job.” Her total score on the Zung Self-Rating Depression Scale [11] was 57, suggesting that her mood had reached the level of neurotic depression or MDD. However, despite her strong subjective depression, she exhibited no sleep or appetite disturbance. Moreover, she said that she was able to enjoy her favorite activities, such as jogging.

*Diagnosis*. Her symptoms did not meet any DSM-IV-TR criteria, including those for MDD. She did show the typical symptoms of MTD; that is, she believed herself to have a strongly depressive mood, even though she could enjoy everything in her life except her job. Furthermore, her main request was not treatment for her depression but rather a medical certificate for a leave of absence. She had never worked hard and exhibited a childish omnipotence in relation to her work; that is, it did not seem necessary for her to work hard because she had sufficient ability. She blamed her boss as if he was interfering with her work. Thus, the doctor judged her to be a typical MTD case.

*In the First Session of IPC*. In a careful and polite tone, the doctor explained to her the following. First, it was no wonder that she had self-diagnosed herself with depression based on Internet information, as there is so much misleading information present on the Internet. Based on a more professional diagnosis, she was not suffering from MDD, a type of psychiatric disorder, but rather MTD, which is a type of depressive situation. Furthermore, a major cause of this situation is a distortion of interpersonal relations. MTD was natural response for young adults in her situation, namely, difficult work and a new boss whom they had never dealt with before. All of these explanations were based on the techniques derived from IPT and are usually used in IPC, namely, explaining the disease and giving the person the role of a sick person. The doctor continued on to explain the treatment of MTD. Particularly, antidepressants are usually ineffective and psychosocial interventions should be primarily considered; however, psychotherapies requiring insight are typically not recommended for treatment. Accordingly, IPC is recommended as the first-line treatment for MTD. The doctor then outlined IPC to her: specifically, it would be provided in 20-minute sessions given essentially every week by the doctor, for a total of 6 sessions. The doctor added that IPC could be done as part of normal medical care and required no additional fees.

She accepted this diagnosis and withdrew her request for a medical certificate of MDD. She appeared to be pleased to hear that she was not a psychiatric patient but rather somebody experiencing a type of depressive mood. She seemed convinced by the explanation that she was not responsible, and that it is common for young adults in her situation to experience a depressive mood. She also seemed satisfied with the doctor's recommendation for counseling without additional charges for that counseling. She said that she felt that she could continue her job if she received counseling, and that she was willing to undergo IPC.

At the end of the session, the problem area was tentatively set as “interpersonal disputes” and/or “role transitions” because she had problems with her boss and her depression had started when she transferred to a new workplace. The doctor asked her to look over recent life events and think again about what was related to her stress reaction.

*In the Second Session of IPC*. She did not mention her depression in the second session. Instead, she talked about how she had enjoyed a temporary paid holiday, which she was able to get without a medical certificate. She further said that she was happy because she had had no troubles at work and had not needed to see her boss during that period. The doctor appreciated her attempts to get a paid holiday instead of taking sick leave. The problem area was discussed and finalized as “interpersonal disputes” with her boss because she felt that the most painful aspect of her situation was not the difficulty of her work in the new workplace but rather the attitude of her new boss.

*In the Third Session of IPC*. She expressed dissatisfaction with her boss, saying “He should have sensed my painful feelings and helped me even if I did not say anything.” The doctor suggested that she address her boss verbally whenever she experienced trouble with him, explaining that her boss likely would not understand unless she said what she felt or thought aloud. The patient looked surprised to hear that; it seemed to be the first time this idea had occurred to her. Additionally, she might have realized that her current communication with her boss was not common in the workplace and that changing her communication pattern would be useful. The doctor went on to point out that this stage of discordance with her boss was renegotiable and gave her homework to report to the boss verbally, regardless of whether her job was going well or not.

*In the Fourth Session of IPC*. She said that she had directly faced her boss. Specifically, after the third session, she had encountered major problems at work, which caused her to become so depressive that she wanted to quit her job. She then told her boss of this desire to quit because her work was so painful and prepared for a reprimand. However, contrary to her expectations, her boss never got angry; instead, he simply said, “Please take a leave of absence from your job for a while.” In hearing her boss say that, she said she suddenly felt relieved and her desire to quit disappeared. The doctor acknowledged her successful experience and encouraged her to continue to engage in communication of a similar degree with her boss.

*In the Fifth Session of IPC*. She said that she was not depressive and was doing well at work. The doctor once again praised her successful experience in the fourth session and encouraged her to engage in further communication.

*In the Sixth Session of IPC*. She reported that she had been doing well. The doctor suggested that she terminate IPC, as she had successfully obtained the ability to communicate with her boss. However, she said that she would like to undergo more sessions because she did not feel sufficiently confident. Therefore, the doctor proposed follow-up sessions but limited these sessions to only two in order to avoid cultivating dependency in the client for the unconditional warmth offered by IPC. She seemed to be somewhat unsatisfied with the doctor's proposal, but she agreed to the follow-up sessions.

*In the Seventh and Eighth Sessions of IPC*. In the seventh session, she felt worried about accepting an offer from her boss. He had asked her to continue the work that she had been doing until that point for the next year, as her boss considered her sufficiently skilled in that work. She did not want to continue with it but felt that she could not say “no” because she was so afraid of losing her boss's trust and high appraisal. She said, “I know I should talk with my boss, but I do not know what to say. I want to quit my job to avoid seeing my boss.” The doctor supported her by explaining that this was a natural feeling and then performed a role-play of a scene where she would inform her boss that she did not want to continue with her job. She said, “It's a very challenging thing, but I'll try.” In the eighth session, she said proudly that she had talked with her boss and that it had gone well. She did not have to continue her work any longer. She said, “I have built up sufficient self-confidence to communicate with my boss through this event.” I proposed that it was perhaps best to terminate the IPC, which she agreed to. Throughout all eight sessions, she was cooperative and enjoyed the counseling. Since then, she has been working without depression for around 2 years.

## 3. Discussion

We present a successful case of IPC for a patient with MTD. This is the first case report suggesting that IPC would be effective for MTD. The IPC helped her to change her pattern of interpersonal relations with her boss, which led to improvement of not only her depressive mood but also her adaptation to her workplace. Consequently, IPC prevented her from further absence or resignation from her work.

We diagnosed the patient with typical MTD using the proposed research-based operational criteria [2]. When assessing MTD, it is important to be cautious—MTD is often misdiagnosed as MDD and treated using antidepressants and rest, especially in primary care. However, such treatment does not have much effect on individuals with MTD and therefore can lead the individual to experience long-term absences from work. Furthermore, even after returning to work, it is common for those with MTD to quickly take another leave of absence. In this case, we did not recommend antidepressants and rest and instead asked the patient to participate in IPC. This helped her to recover gradually from the disease without taking a leave of absence or quitting outright.

MTD is often comorbid with a personality disorder [2]. However, the case described in this study had no such disorder based on DSM-IV-TR criteria, despite her childish attitude and somewhat narcissistic and immature personality. Actually, these characteristics are rather common among Japanese young adults nowadays [2]. As often seen among individuals with MTD, she blamed her boss for her depression possibly because she was unconsciously afraid that her boss would hurt her pride by pointing out that she did not have sufficient ability to perform her work. On the other hand, she was cooperative and far from being extrapunitive throughout the sessions. The unconditional warmth of the IPC might have encouraged this attitude. Based on our results, MTD seems receptive to treatment with IPC.

Another reason that IPC might be useful for MTD is its educational aspect. This case had exceedingly immature communication skills, such that she believed help would be provided by others, especially her social superiors, without her asking for it. Through IPC, she learned the communications skills appropriate for interacting with her boss. It is not likely that her communication with all other individuals improved. Instead, it probably only improved for that particular situation that had depressed her. Given the expected long-term effects of IPC, however, it might be that she could apply the skills learned throughout her eight sessions of IPC to other situations in the future. In fact, such long-term effects appear supported by our findings, as the woman has been working without depression for around 2 years.

The low burden of IPC, on both patients and doctors, might be another reason for its effectiveness for MTD. If a patient can receive IPC without much time or economic burden, their motivation to receive counseling might increase, along with the treatment effects. It also has high cost-effectiveness for use in primary care, which may explain its efficacy for treating patients with MTD.

Although we attempted to perform IPC in line with the IPC manual in Japanese [9], this was not entirely possible. First, in general, the IPC manual limits the number of sessions to six in order to avoid cultivating excessive dependence in the patient. However, we performed eight sessions. As the IPC manual does allow for more than two follow-up sessions, depending on patients' need, the two follow-up sessions in our study are not a major methodological problem. Nevertheless, there still might be a risk of cultivating excessive dependency in the patient. Second, the IPC manual recommends weekly sessions, but the interval between sessions in this case was occasionally as long as a month because of the need to accommodate the patient's schedule. However, it still showed a good effect. This longer interval could have given the patient time to practice communication methods introduced in the counseling. Thus, conducting IPC with a greater degree of flexibility than the procedure outlined in the manual might be useful for primary care in Japan. It should be noted that the newly released IPC manual has reduced the number of sessions to three [12–14]. These new IPC manuals might be much more convenient in general practice in Japan, as the fewer sessions of IPC could better fit into patients' schedules.

This study contains several limitations. First, it was a case report, so we cannot generalize the results. Second, we could not perform the IPC completely in line with the manual. Nevertheless, the results suggest that IPC could be effective for treating MTD. Further studies should be done in the future.

## 4. Conclusion

We outlined a case with MTD for whom IPC was effective. The IPC improved the patient's communication and depressive mood. IPC might be useful in primary care for the treatment of MTD and could be one way of preventing young workers from easily resigning from their jobs as a result of depression.
